# Development of Virus-Like Particles (VLPs) for Hepatitis C Virus genotype 4: a novel approach for vaccine development in Egypt

**DOI:** 10.1186/s12896-024-00935-5

**Published:** 2025-01-18

**Authors:** Ahmed A. Ali, Rasha A. M. Azouz, Nahla A. Hussein, Reem El-Shenawy, Naiera M. Helmy, Yasmine S. El-Abd, Ashraf A. Tabll

**Affiliations:** 1 Molecular Biology Department, Biotechnology Research Institute, National Research Centre, Cairo, 12622 Egypt; 2 Microbial Biotechnology Department, Biotechnology Research Institute, National Research Centre, Cairo, 12622 Egypt; 3 Egyptian Centre for Research and Regenerative Medicine (ECRRM), Cairo, 11517 Egypt

**Keywords:** Hepatitis C Virus (HCV), Virus-Like Particles (VLPs), Genotype 4, Structural proteins, Vaccine, Lentivectors, Stable cells

## Abstract

**Background:**

Egypt has the highest global prevalence of Hepatitis C Virus (HCV) infection, particularly of genotype 4. The development of a prophylactic vaccine remains crucial for HCV eradication, yet no such vaccine currently exists due to the vaccine development challenges. The ability of Virus-Like Particles (VLPs) to mimic the native virus and incorporate neutralizing and conformational epitopes, while effectively engaging both humoral and cellular immune responses, makes them a promising approach to addressing the challenges in HCV vaccine development.

**Methods:**

Lentiviral-based vectors were constructed and employed to integrate the full-length sequence of Core, E1, E2, and P7 genes of HCV genotype 4 into the genome of Human Embryonic Kidney cells (HEK293T). Upon the expression, HCV structural proteins can oligomerize and self-assemble into VLPs mimicking the structure of HCV native virus. VLPs were purified and characterized for the development of a potential VLPs-based vaccine.

**Results:**

In this study, mammalian cells were successfully engineered to stably express HCV structural proteins and generate non-infectious VLPs for HCV genotype 4. The expression of HCV-integrated genes resulted in a successful production of HCV structural proteins, which oligomerized and self-assembled into two layers enveloped VLPs. Electron microscopy analysis of purified VLPs revealed spherical particles with an average diameter of 60–65 nm, closely resembling mature HCV virions. These results highlighted the potential of these VLPs as a vaccine candidate for HCV genotype 4.

**Conclusions:**

HCV genotype 4 remains an underexplored target in vaccine development, despite its significant public health burden, especially in Egypt. The successful generation of VLPs for this genotype represents a promising avenue for further vaccine development. The established system provides a robust platform for the production and study of VLP-based vaccines targeting HCV genotype 4.

## Introduction

Hepatitis C Virus (HCV) is a bloodborne pathogen that has chronically infected 50 million people worldwide, with approximately 1 million new infections every year. Most of cases develop chronic infections whereas 20% of patients develop long-term complications such as liver fibrosis, cirrhosis and hepatocellular carcinoma (HCC). Globally, HCV infection and its complications cause 242,000 deaths annually. Only 20% of HCV-infected patients are diagnosed and 15% of those have been treated with the Direct Acting Antiviral Drugs (DAAs) [1, 2]. Egypt has the highest prevalence rate of HCV infection in the world [3–5]. According to the latest Egyptian Health Issues Survey (EHIS) which was conducted on behalf of the Ministry of Health and Population in 2015, approximately 6 million Egyptians are infected with HCV, representing about 7.0% to 10% of the country’s total population with 150,000 new infections every year. Eighty percent of those infected develop chronic HCV which can lead to liver cirrhosis and cause most of HCC cases in Egypt [6–8].

In 2014, the Egyptian government launched an extensive HCV management program using DAAs to eliminate HCV infection nationwide [8, 9]. This program has significantly reduced the number of HCV-infected individuals and HCV-related complications. However, substantial efforts are still needed to achieve the full HCV elimination and overcome the remaining challenges of DAAs such as the limited access to diagnosis and treatment, low follow-up rates, high reinfection rates, and the resistance to DAAs treatment [10–14]. Additionally, the high cost of these medications restricts availability in low and middle-income countries [11, 15–17]. Thus, long-term protection and prevention of chronic HCV infection require developing a cost-effective prophylactic vaccine to prevent the transmission of the virus and protect the high risk groups [18–22].

Globally, HCV has been classified into 8 genotypes and 93 subtypes with differences of approximately 30% in the genome sequence [23, 24]. HCV genotype 4 is mainly prevalent in the Middle East and Africa. In Egypt, HCV genotype 4 is the most dominant genotype and accounts for 90% of total HCV infections [7]. Many trials have been ongoing for decades to develop an effective prophylactic vaccine for HCV but yet there is no approved vaccine. The development of an effective vaccine for HCV is a challenging process due to the virus’s high genetic variability, numerous viral evasion strategies, lack of a reliable animal model, incomplete understanding of protective immune responses, the long duration of chronic infection, and difficulties in performing clinical trials [11, 25–27].

Virus-like particles, VLPs, are very promising platform for production of effective vaccines that can overcome many challenges in HCV vaccine development [28]. VLPs are nanoparticles composed of one or more viral structural proteins that can spontaneously self-assemble to closely mimic the structure of the native virus but without the infectious genome. They can present multiple antigens in the correct conformation displaying many neutralizing and conformational structure-dependent epitopes. VLP-based vaccines are safe and potent immunogens that can efficiently stimulate both cellular and humoral immune responses [29–31]. Mammalian cells, being natural hosts for human viruses, are considered the optimal expression system for VLPs production due to their capability to perform complex post-translational modifications such as glycosylation patterns, which is crucial for the correct folding and assembly of VLPs, enabling the assembly of authentic VLPs that closely resemble the native virus [32].

The VLPs-based technology was used to develop safe and effective vaccines against some viral infections. The best examples are the approved VLPs-based vaccines that proved a great success in protecting against some viral infections such as GSK Engerix and Merck Recombivax-HB for hepatitis B virus and GSK Cervarix and Merck Gardasil for human papillomavirus, HPV [33, 34]. Therefore, the development of VLPs for HCV as a potential vaccine candidate could solve many problems that face HCV vaccine development. Numerous VLPs developed for some HCV genotypes like genotypes 1, 2, 3, and 6 in different expression systems demonstrated strong humoral and cellular immune responses [29, 35–40]. However, VLPs for HCV genotype 4, which is the most prevalent genotype in Egypt, have not yet been generated or evaluated as a potential vaccine candidate. Therefore, developing VLPs as a potential VLPs-based vaccine candidate to target this specific genotype could have a significant impact on the virus transmission and progression to chronic infection in Egypt and similar countries. Additionally, the development of stable mammalian cell line for persistent expression of HCV structural proteins and consistent generation of VLPs will increase the yield and reduce the cost of vaccine production [41].

In this study, we successfully developed a mammalian cell line capable of stably expressing HCV structural proteins (Core, E1, E2 and P7) that can oligomerize and self-assemble into VLPs for HCV genotype 4. The generated VLPs were shown to be two-layers enveloped particles with a spherical shape and an average size of 60–65 nm. The produced VLPs by presenting all the viral structural proteins’ epitopes in their proper conformation as the native virus, are a promising platform for production of a potential vaccine for HCV genotype 4. The constructed VLPs can also serve as a safe non-infectious model to study the virus entry, assembly and virus-host interactions for this genotype without the need for handling the infectious virus. In addition, the established stable cell line with good expression levels of HCV structural proteins and VLPs can be used to develop cost-effective HCV-specific diagnostic assays and reagents.

## Materials and methods

### Plasmid construction

To generate a Lentiviral Transfer Vector encoding HCV (genotype 4a) structural genes (pLV-HCV4a C–P7), a single ORF DNA fragment comprising the sequence of Core, Envelope 1 (E1), Envelope 2 (E2) and P7 ORFs of HCV genotype 4a (Protein ID: ADF97233.1, GenBank: GU814265.1) was synthesized and cloned into a third generation lentiviral transfer vector (pLV, VectorBuilder Inc, USA) under the control of EF1α mammalian promoter and in fusion with 10x His-tag at the N-terminus of the HCV Core sequence. The ORFs sequence of HCV genotype 4a was sourced from the published full-length cDNA sequence of HCV genotype 4a, which was isolated from an Egyptian patient [42, 43]. The antibiotic resistance gene, Puromycin, was incorporated into the constructed plasmid to facilitate the selection of infected cells. The lentiviral transfer, packaging, and envelope plasmids, pWPXL, psPAX2, and pMD2G, respectively were kindly provided by Dr. Didier Trono (EPFL, Lausanne, Switzerland). High quality plasmids’ DNA was prepared for cell transfection and lentiviruses production using PurYield Maxiprep Kit (Promega, USA).

### Cell culture

Human Embryonic Kidney Cells, HEK293T (kindly provided by Dr Martin Schroeder, Department of Biosciences, Durham University, UK) were cultured at 37 °C with 5% CO_2_ in CO_2_ incubator (Series 8000 Water-Jacketed CO_2_ Incubator, ThermoFisher Scientific, USA) in complete Dulbecco’s Modified Eagle Medium (DMEM) (Gibco, Invitrogen) with 10% heat-inactivated Fetal Bovine Serum, FBS (Gibco, Invitrogen), 100 U/ml Penicillin and 100 μg/ml Streptomycin antibiotics, 1 mM sodium pyruvate, 2 mM L-Glutamine, and 1% (w/v) Non- Essential Amino Acids (NEAA).

### Cells transfection

The transfection of HEK293T cells was conducted by the Calcium Phosphate Precipitation Method as described by Kriegler [44]. Briefly, HEK293T cells were seeded in a suitable culture dish or plate at 37 °C and 5% CO_2_ for 24 h until cells reached 70–80% confluence on the transfection day. After 24 h, the medium was replaced with fresh complete DMEM medium two-hours before the transfection. For a well in a 6-well plate, 10–15 μg of high-purity plasmid DNA was diluted in sterile dH_2_O to a final volume of 250 μl. An equal volume (250 μl) of 2x HEPES Buffered Saline (2X HBS, 50 mM HEPES, pH 7.15, 280 mM NaCl, 1.5 mM Na_2_HPO_4_) was added to the DNA and mixed well before the dropwise addition of 25 μl of 2.5 M CaCl_2_ (Sigma, USA). The DNA/phosphate mixture was incubated at room temperature for 30 min before dispersing the mixture on cells by gentle swirling. The cells were then incubated at 37 °C and 5% CO_2_ for 24 h. The next day, the transfection medium was exchanged with fresh complete DMEM medium containing 1 mM sodium butyrate (Sigma, USA) and incubated for 24 h at 37 °C and 5% CO_2_. The gene expression was investigated 48 h post-transfection by ELISA, western blotting, fluorescence and/or immunofluorescence using the Zoe Fluorescent Cell Imager (BioRad, USA).

### Lentiviruses (LVs) production

Lentiviruses production was conducted in HEK293T as packaging cells following the previously described method with some adjustments [45]. Cells were plated in 60 mm cell culture dishes one day before transfection and incubated at 37 °C and 5% CO_2_ for 24 h. The following day, the medium was replaced two hours before transfection with a fresh complete DMEM medium. In a sterile Eppendorf tube, 20 μg DNA of HCV transfer vector (pLV-HCV4a C–P7) or EGFP control vector (pWPXL), along with 20 μg of packaging plasmid (psPAX2), and 10 μg of envelope plasmid (pMD2.G), were combined in a ratio of 2:2:1, respectively. Sterile distilled H_2_O was added to the total DNA mixture to a final volume of 300 μl. An equal volume (300 μl) of 2X HBS (pH 7.15) was added to the DNA mixture and mixed thoroughly before the dropwise addition of 30 μl of 2.5 M CaCl_2_ (Sigma, USA) with continuous mixing by vortex. The DNA/phosphate mixture was then incubated at room temperature for 30 min. After the incubation, the DNA precipitates suspension was gently vortexed and added dropwisely to the cells with good mixing by swirling. The cells were then incubated at 37 °C and 5% CO_2_ for 24 h. The next day, the medium was replaced with low-serum DMEM (2% FBS) containing 1 mM sodium butyrate (Sigma, USA) and incubated at 37 °C and 5% CO_2_ for an additional 24-hours before the first collection of the virus-containing medium. The subsequent harvesting of the virus-containing media was made at 72- and 96-hours post-transfection. The collected media from all harvests were combined and cleared by low-speed centrifugation at 1500 rpm for 5 min at 4 °C, followed by passage through a 0.45 μm sterile syringe filter (Millipore, USA). The virus-containing media could be used directly to infect cells or stored at −80 °C in aliquots until needed. When necessary, the virus-containing medium was concentrated using Vivaspin Centrifugal Filters, 100kDa MWCO (Sartorius, Germany) or by ultracentrifugation for 120 min at 50,000×*g* at 4 °C. The supernatant was discarded, and the virus pellet was resuspended in PBS and stored in aliquots at −80 °C.

### Cells transduction

The lentiviral transduction of target cells (HEK293T) was performed by resuspending 1 × 10^6^ of cells pellet in 1 ml of lentiviruses-containing medium at a multiplicity of infection (MOI) of 2 (2 VLPs:1 Cells) in the presence of 8 μg/ml Polybrene. Cells were incubated with the virus for 20 min at 37 °C. After incubation, cells were cultured in 60 cm culture dish in a Low-serum DMEM medium containing 8 μg/ml polybrene and incubated at 37 °C and 5% CO_2_ for 24 h. The next day, the virus-containing medium was replaced with a fresh complete DMEM medium and incubated for another 24 h at 37 °C with 5% CO_2_. 48 h-post-transduction, the infection efficiency was evaluated by examining the expression of EGFP of control plasmid (pWPXL), flow cytometry and/or immunofluorescence staining of HCV core protein. For isolation of stably infected cells, transduced cells were further propagated in complete DMEM medium (10% FBS) containing 1 μg/ml Puromycin antibiotic (Sigma, USA) for 4–5 days at 37 °C and 5% CO_2_. During the antibiotic incubation time, suspended and dead cells were discarded from the dish. After the incubation, fresh complete DMEM medium without the antibiotic was added to the infected cells for cells recovery. The survived infected cells were then maintained in complete DMEM for recovery and amplification. Cells were then stored in aliquots in freezing medium (10% DMSO) in −80 °C freezer or liquid nitrogen.

### Lentivectors (LVs) titration

The functional Lentiviruses (LVs) titers were determined by either serial dilution infection or flow cytometric analysis of EGFP expression in the infected HEK293T cells using the control lentiviral transfer plasmid (pWPXL). Briefly, HEK293T cells were seeded in 24-well plate to reach about 80% at the infection time. The next day, the medium was changed with fresh low-serum DMEM medium containing polybrene at a final concentration of 8 μg/mL. The cells were infected with serial dilutions of HCV4a-Lentiviruses- or EGFP-Lentiviruses-containing media in the presence of polybrene (8 μg/ml) and incubated for 24 h at 37 °C and 5% CO_2_. The following day, the infection medium was replaced with fresh complete DMEM and further incubated for 24 h at the same conditions. The cells were examined by the Zoe Fluorescent Cell Imager (BioRad, USA) 48 h post-infection for the EGFP expression by counting the number of fluorescent cells in wells infected with the lowest viral dilution and multiplying in the dilution factor to determine the number of LVs transducing units per milliliter (TU/ml). The EGFP fluorescence of EGFP-LVs-infected cells was also analyzed by the flow cytometry 48 h- post-infection to count the number of infected fluorescent cells compared with the total number of cells. HCV4a-Lentiviruses titration was performed by the same serial dilution way, but the efficiency of infection was investigated by immunostaining of the HCV Core protein using HCV specific anti-core antibodies and secondary antibodies conjugated with Alexa Fluor 555. The fluorescence of HCV4a-Lentiviruses-infected cells was investigated by the red channel of the Zoe Fluorescent Cell Imager (BioRad, USA).

### Flow cytometry

The flow cytometric analysis was conducted for titrating the number of EGFP-LVs produced in the packaging cells. Briefly, HEK293T cells were cultured in 6-well plates at a density of 5 × 10^5^ cells/well and incubated overnight at 37 °C with 5% CO_2_ to reach approximately 50–60% confluence by the following day. The cells were infected with EGFP-LVs using different viral dilutions of the virus-containing medium with 8 μg/ml polybrene. The infected cells were incubated for 24 hours at 37 °C and 5% CO_2_. Subsequently, the virus-containing medium was replaced with fresh complete DMEM medium, and cells were incubated under the same conditions for an additional 24 h. 48 hours post-transduction, cells were washed with PBS, harvested, and transferred to labeled 15-ml Falcon tubes. The cells were centrifuged at 1000 rpm and the cell pellet was resuspended in 0.5 ml of fresh PBS for FACS analysis. Non-infected cells were used as a negative control. The FACS analysis was conducted by Flow Cytometry (CytoFlex, Beckman Coulter) to determine the percentage of EGFP-positive cells, with cells exhibiting 10–20% positivity selected for titer calculation using the specific equation; Lentiviruses Titer (TU/ml) = (N × P)/(V × D), where N = Cell Number in each well used for infection, P = Percentage of EGFP-positive cells, V = Lentiviruses volume used for infection in each well, D = Dilution fold, TU = Transduction Unit.

### HCV4a genes’ integration into the host cell genome

To investigate the integration of HCV structural genes (HCV Core, E1, E2 and P7) into the genome of produced HCV4a-HEK293T stable cell line, the cells were cultured in 6-well plate at 200,000 cells/well. The following day, the total genomic DNA was isolated from infected cells using the TRIzol Reagent (ThermoFisher Scientific, USA) according to the manufacturer’s instructions. The concentration of extracted genomic DNA was measured at 260 nm using a NanoDrop spectrophotometer (ThermoFisher Scientific, USA). The total genomic DNA was used as a template for Polymerase Chain Reaction (PCR) to detect the presence of HCV genes in the genome of infected and stable cells using the specific primers for HCV genotype 4a structural genes listed in Table 1.

**Table 1:** The list of primers used in this study

Primer	Sequence
GT4-Core-F	CCTAACGCGTACCATGAGCACGAATCCTAAACC
GT4-E2–R	GCCGACTAGTTTACGCCTCAACTTGACTTACC
pWPXL-Seq-For	AGCAACAGACATACAAAC
pWPXL-Seq-Rev	CATAGCGTAAAAGGAGCAAC
HCV4a-RT-Core-F	ATGAGCACGAATCCTAAACCTC
HCV4a-RT-Core-R	TGGATATCCTGGTTGTGCCC
HCV4a-RT–E1–F	CGCAATGTCTCGGGCATCTA
HCV4a-RT–E1–R	CCCCCACCATCAAATCCACA
HCV4a-RT–E2–F	GAGACTCACGTGTCTGGGG
HCV4a-RT–E2–R	CCGTAGCTGTCAAGGCTCTT
Human β-Actin-F	CACCAACTGGGACGACAT
Human β-Actin-R	ACAGCCTGGATAGCAACG

A list of primers used in the PCR analysis to amplify and detect the EGFP, HCV4a Core, E1 or E2 and β-Actin genes sequences for the integration and mRNA expression protocols

The PCR was performed using the Phusion Hot Start II High-Fidelity or *Taq* DNA Polymerases (ThermoFisher Scientific, USA) according to the manufacturer’s instructions. The amplified DNA fragments were visualized and analysed by resolving the PCR products on 1% agarose gel in TAE running buffer for 40 min at 100 Volts (V) using a gated mini-gel tank (BioRad, USA). GeneRuler 1kb DNA Ladder (ThermoFisher Scientific, USA) was used as a molecular weight standard. The DNA bands on the gel was visualized by a UV transilluminator equipped with a camera (Syngene) to capture images.

### The mRNA expression of HCV4a-integrated genes

To investigate the mRNA expression of HCV-integrated genes in HCV4a-HEK293T stable cells, the total RNA was isolated from HEK293T control and HCV4a-HEK293T stable cells using TRIzol reagent (ThermoFisher Scientific, USA) following the manufacturer’s instructions. The extracted RNA was used as a template for cDNA synthesis for both samples using the High-Capacity cDNA Reverse Transcription Kit (ThermoFisher Scientific, USA). The synthesized cDNA of both samples was subjected to a PCR analysis using forward and reverse primers specific to each HCV4a gene where HCV4a-RT-Core-F and HCV4a-RT-Core-R were used to amplify a fragment of 245 bp of the core gene, HCV4a-RT–E1–F and HCV4a-RT–E1–R for amplification of 219 bp of the E1 gene, while HCV4a-RT–E2–F and HCV4a-RT–E2–R to amplify a fragment of 247 bp of the E2 gene (Table 1). The Human β-Actin-F and Human β-Actin-R primers were used to amplify a fragment of β-actin gene as a control housekeeping gene. The PCR composition and conditions were optimized and performed according to the *Taq* DNA Polymerase kit instructions (ThermoFisher Scientific, USA).

### Immunofluorescence staining (IF)

The immunofluorescence (IF) assay was conducted in a 24-well plate 48 h post-transfection, -infection, or for stable cells to detect the expression of HCV core protein. In brief, transfected, infected, or stable HCV4a-HEK293T cells were cultured and incubated for 48 h at 37 °C and 5% CO_2_. After incubation, the medium was aspirated from wells, and cells were briefly rinsed with PBS. Subsequently, the cells were fixed in 300 µl of cold 100% methanol (pre-chilled at −20 °C) for 5 min at room temperature. Following fixation, cells were washed three times with PBS and incubated in a blocking solution (1% BSA, 22.52 mg/mL glycine) made in PBST (PBS+ 0.1% Tween 20) for 30 min at room temperature to prevent nonspecific binding of antibodies. Afterward, the cells were washed with PBS three times and incubated with primary antibodies at the recommended dilution (1:3000 dilution for the HCV core antigen mouse monoclonal antibodies, C7-50, ThermoFisher Scientific, USA) in PBST containing 0.1% BSA for 1 hour at room temperature or overnight at 4 °C. Following the primary antibodies incubation, cells were washed with PBS and incubated in the dark with the goat anti-mouse Alexa Fluor 555 secondary antibodies, ThermoFisher Scientific, USA (4 µg/ml in PBST with 0.1% BSA) for 1 hour at room temperature. The cells were washed with PBS and the cell nuclei were counterstained in the dark with the Hoechst stain dye 33342 (ThermoFisher Scientific, USA) at a concentration of 0.1–1 μg/mL for 5 min at room temperature. The cells were rinsed with PBS, and the fluorescence analysis was conducted using the Zoe Fluorescent Cell Imager (BioRad, USA).

### Proteinase K protection assay

The proteinase K protection assay was performed to investigate the formation of VLPs for HCV genotype 4 in the developed HCV4a-HEK293T stable cells according to the previously described method [46]. Briefly, the stable cells were cultured in two 150 mm cell culture dishes in complete DMEM for 48 h at 37 °C and 5% CO_2_. Subsequently, the medium was aspirated from the dishes and the cells were detached by pipetting up and down in PBS before transferring them into 15 mL Falcon tubes. The cells were then collected by low-speed centrifugation at 1500 rpm for 5 min at 4 °C. The cells pellet was resuspended in PBS and centrifuged again to pellet the cells. The PBS was aspirated, and the cell pellet was resuspended in an ice-cold proteinase K buffer (50 mM Tris/HCl pH 8, 10 mM CaCl_2_, 1 mM DTT) and incubated on ice or frozen at −80 °C until further processing. The cells were subjected to multiple freezing/thawing cycles at −80 °C to for cell lysis before dividing the cleared lysate into three equal parts in new microfuge tubes, with specific treatment conditions applied as per the following: Tube 1 (no treatment), Tube 2 (treated with 5 μl of 10% Triton X100 for 5 min on ice followed by the treatment with 2 μl of Proteinase K solution (1.25 mg/ml) for 30 min on ice) and Tube 3 (treated with 2 μl of Proteinase K solution (1.25 mg/ml) for 30 min on ice). After the proteinase k treatment, 2 μl of PMSF (5 mg/ml) was added to Tube 2 and 3 and incubated on ice for 10 min to stop the proteinase k activity. Following the treatments, 6x SDS buffer was added to each tube, and the samples were used immediately for 10%SDS-PAGE and western blotting analysis. Subsequently, samples were subjected to 10% SDS-PAGE and western blotting analysis to detect the HCV Core protein using the HCV core antigen mouse monoclonal primary antibodies (C7-50) and the goat anti-mouse-HRP secondary antibodies.

### Western blotting (WB) analysis

The western blotting (WB) was used to detect the expression of HCV Core protein with specific antibodies. Protein samples were prepared by mixing them with 6x SDS sample buffer (0.125 M Tris-HCl (pH 6.8), 4% SDS, 10% β-Mercaptoethanol, 20% Glycerol, 20 mg Bromophenol Blue) and boiling at 95 °C for 5 min. The proteins were then separated using 10% SDS-PAGE in 1X Tris-Glycine running buffer, using BioRad Proteon II minigel apparatus following the manufacturer’s instructions. Electrophoresis was performed initially at 50 V for 10 min, then at 150 V for 1 hour, or until the bromophenol blue dye reached the gel’s bottom. The PageRuler Pre-Stained Protein Ladder (ThermoFisher Scientific, USA) was used as a guide for protein size. After electrophoresis, the gel was either stained with Coomassie blue to visualize the protein bands or transferred to a nitrocellulose or PVDF membrane (ThermoFisher Scientific, USA) for western blotting analysis.

Proteins were transferred from the SDS-PAGE gel to an PVDF membrane using a semi-dry transfer apparatus (BioRad, USA) at 15 V for 30 min. The membrane was then blocked with 5% non-fat dry milk in TBST for 1 hour at room temperature to prevent non-specific binding. After blocking, the membrane was washed three times with TBST, followed by incubation with the HCV core antigen mouse monoclonal antibodies (C7-50) diluted in blocking buffer (1:2000) for 1 hour at room temperature with shaking. After three more washes, the goat anti-mouse IgG (H+L) secondary antibodies (ThermoFisher Scientific, USA) conjugated to the horseradish peroxidase (HRP) was applied at 1:5000 dilution in blocking buffer for 1 hour at room temperature. Following three additional washes with TBST, the protein bands were developed using SuperSignal West Pico PLUS Chemiluminescent Substrate (ThermoFisher Scientific, USA) in a dark room. The membrane was exposed to an X-ray film for 1–5 min, and the film was manually developed using commercial Developer and Fixer solutions.

### Purification of VLPs from HCV4a-HEK293T stable cells

To isolate the generated VLPs from the established HCV4a-HEK293T stable cells, the cells were plated in two 150 mm cell culture dishes for two days at 37 °C and 5% CO_2_ until cells reached 80–90% confluence. Cells were collected and gently lysed in the proteinase k buffer by several cycles of freezing and thawing at −80 °C or Liquid N_2_. After clearing the lysate by low-speed centrifugation (5000 rpm for 15 min at room temperature), the lysate was laid onto the top of 30–50 ml sucrose cushion (30% in PBS) and subjected to ultracentrifugation for 2 h at 40,000 rpm at 4 °C. The VLPs-containing pellet was resuspended in PBS and stored in aliquots at −80 °C until use.

### Enzyme-linked immune sorbent assay (ELISA)

An ELISA assay was conducted to investigate the presence of HCV Core protein in the stable cells and the purified VLPs, prepared from two 150 mm cell culture dishes at 80–90% confluence. U-shaped 96-well polyvinyl plates were first coated with purified VLPs or HCV4a-HEK293T cell lysate (35 µg/well) in a carbonate coating buffer (100 mM Na_2_CO_3_ and NaHCO_3_, pH 9.6) and incubated overnight at 4 °C. The wells were washed five times with PBS containing 0.05% Tween 20 (PBST). Non-specific binding was blocked with 5% skimmed milk in PBST for 2 h at room temperature. After washing, wells were incubated with HCV core antigen mouse monoclonal antibodies (C7-50, ThermoFisher Scientific, USA) at 1:1000 dilution for 2 h at room temperature. The plate was washed with PBST and then incubated with goat anti-mouse-HRP secondary antibodies (ThermoFisher Scientific, USA) at 1:5000 dilution in PBST for 1 h at RT. After washing the plate with PBST, the TMB-ELISA substrate (ThermoFisher Scientific, USA) was added and incubated for 15 min at room temperature. The reaction was stopped with 2 M HCL, and absorbance was measured at 450 nm using an ELISA plate reader (Serial Number: 4300-2017, CHROMATE, Model: 4300, Awareness Technology, Inc., Palm City, FL, USA). The amount of fold change of HCV Core protein after ultracentrifugation on 30% sucrose cushion was calculated as the ratio of the Core protein concentration in the purified VLPs pellet to its concentration in the stable cell lysate as an original starting material, (OD450 for Core in VLPs pellet/OD450 of Core in stable cell lysate). This approach provided a good measure of VLPs enrichment, supporting the integrity of our purification process.

### Negative-staining transmission electron microscope (TEM)

To purify and characterize the HCV4a Virus-Like Particles, HCV4a-VLPs, generated by the developed HCV4a-HEK293T stable cells, the HCV4a-VLPs were isolated from the cell lysate on sucrose cushion by ultracentrifugation and investigated by the Transmission Electron Microscope (TEM) at the Electron Microscope Unit, NRC, Egypt. The examination of HCV4a-VLPs was conducted using the negative staining technique with the Phosphotungstic Acid (PTA), as previously described [47]. A few drops of the HCV4a-VLPs suspension were applied to a carbon-coated TEM copper grid and incubated for 10 min in a moist chamber to minimize the evaporation. The excess liquid was carefully removed from the grid’s surface with a filter paper. The grid was then washed three times by gently touching it to drops of deionized water, with the remaining water wicked away each time. A small drop of 1% PTA stain was applied to the grid and left for 10 s to 1 min. After removing the stain by touching the edge of the grid to a filter paper, the grid was air-dried at room temperature and examined using the TEM imaging.

## Results

### Design and construction of lentiviral-based vector for expression of core, E1, E2 and P7 of HCV genotype 4a

**Fig. 1:**
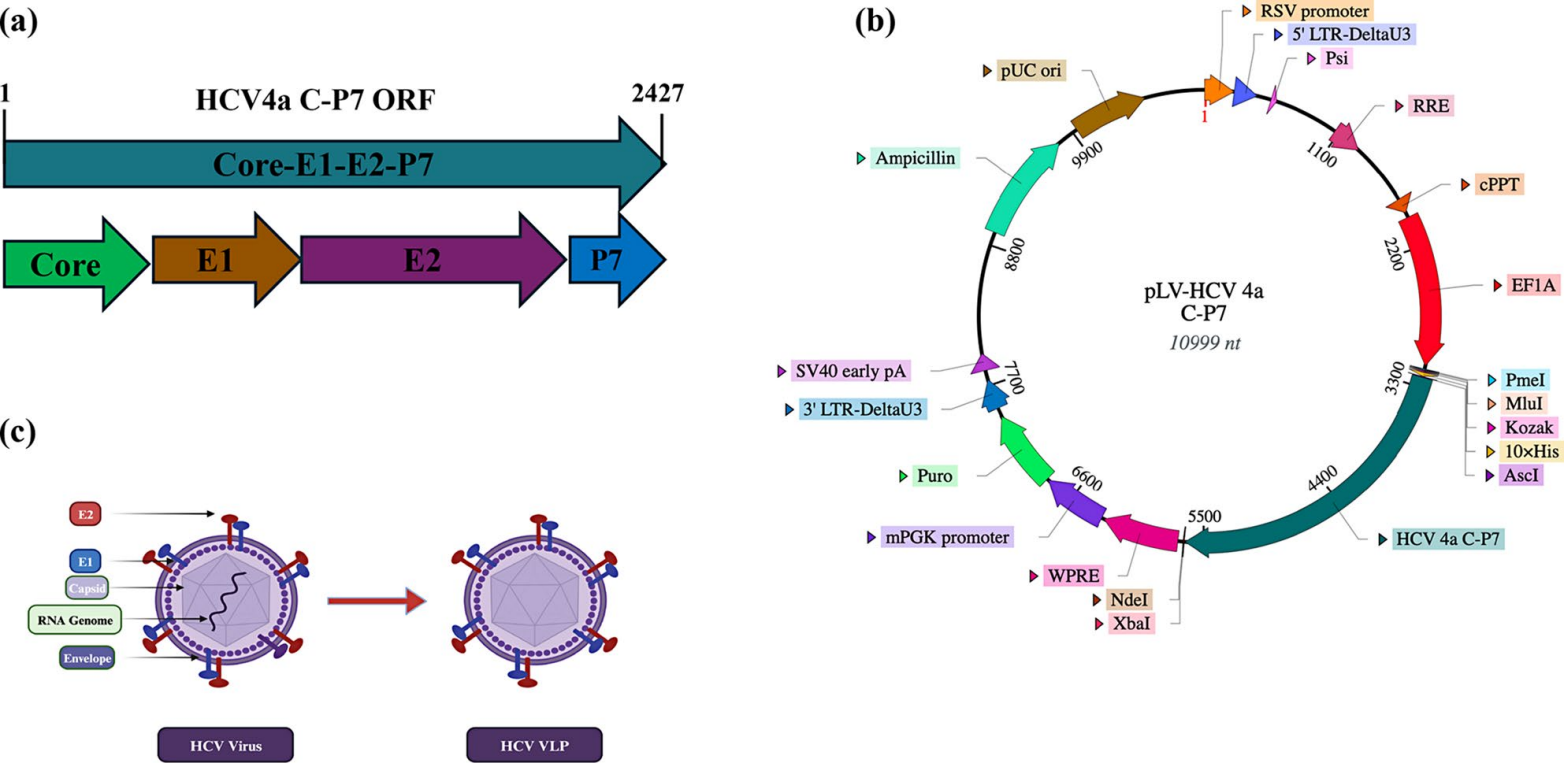
Design and construction of HCV4a lentiviral transfer plasmid, pLV-HCV4a C–P7. Schematic diagrams showing **a** The structure of HCV4a C–P7 ORF cassette encompassing the full cDNA sequence of Core, E1, E2 and P7 ORFs of HCV genotype 4a. **b** A genetic map of the constructed lentiviral transfer plasmid, pLV-HCV4a C–P7, encoding Core, E1, E2 and P7 ORFs of HCV genotype 4a under the control of EF1α mammalian expression promoter and contains puromycin-resistance gene for the selection of stable cells. **c** A schematic diagram showing the structure of HCV native virus where the viral genome is encased in a capsid of the viral core protein which is enveloped by an envelope of a lipid bilayer derived from host membranes and HCV E1 and E2. The diagram is also showing the structure of the Virus-like particle (VLP) which is the same as the native virus but without the infectious genome. This diagram was created with BioRender.com

HCV genotype 4a is the most dominant genotype in Egypt which represents 90% of total HCV infections. Targeting this genotype by an effective vaccination could have a great impact on the clearance of the virus. The design of the lentiviral-based vector was planned to incorporate all the HCV structural genes ORFs (Core, E1 and E2) plus the non-structural gene P7 into a third-generation lentiviral transfer plasmid (Fig. 1a). This system included also all the necessary elements required for transferring and integrating the four HCV genes into the genome of host cells.

To generate a lentiviral transfer plasmid encoding the sequence of HCV genotype 4a structural genes, the full sequence of four ORFs of Core, E1, E2 and P7 of HCV genotype 4a were obtained from the previously published sequence isolated from an Egyptian patient (Protein ID: ADF97233.1, GenBank: GU814265.1). The full sequence of HCV genes was synthesised as a single Core-E1–E2–P7 ORF and cloned into a 3rd Generation Lentivector Transfer Plasmid, pLV (VectorBuilder Inc, USA) under the control of EF1α mammalian promoter and in fusion with 10xHis-tag at the beginning of the ORF to form the lentivector transfer plasmid pLV-HCV4a C–P7 (Fig. 1b). The ORF of P7 sequence was also included with the HCV structural genes to help in the VLPs assembly as previously mentioned in some studies [48]. A puromycin resistance gene was included in the lentiviral transfer plasmid as a selection marker to select the infected stable cells with the HCV genes integrated in their genome. Typically, upon the expression of integrated genes, the HCV structural proteins will oligomerize and self-assemble into VLPs like the actual virus but lacking the infectious viral genome (Fig. 1c). The lentiviral transfer plasmid (pWPXL) was employed in this study as a control for optimization of in vitro transfection, lentiviral production and infection protocols. The obtained plasmid was subjected to DNA sequencing and PCR analysis to confirm its identity.

### Efficient expression of HCV structural proteins by the constructed lentiviral transfer vector

**Fig. 2:**
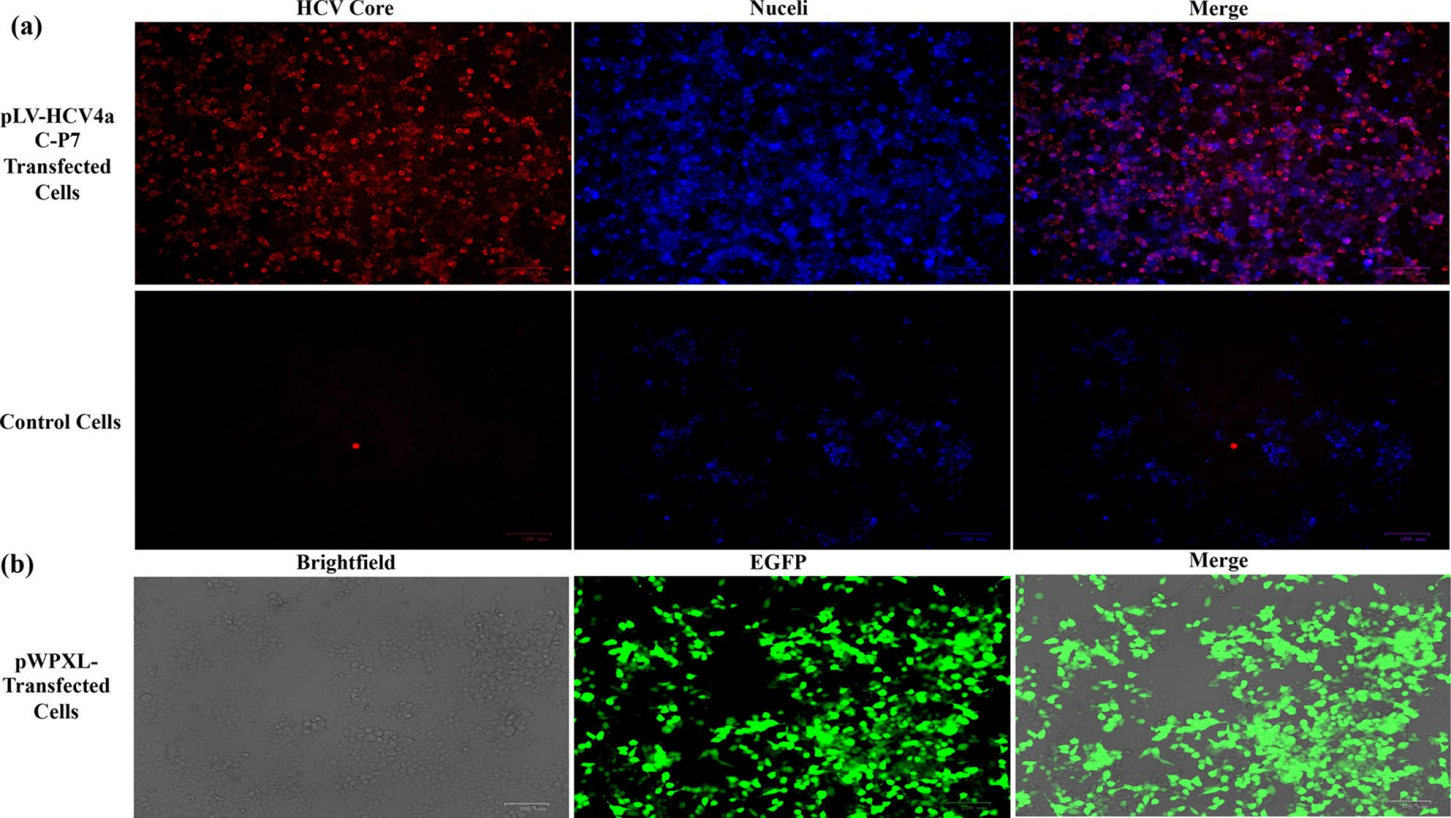
Expression of HCV4a proteins by the lentiviral transfer plasmid, pLV-HCV4a C–P7. HEK293T cells were transfected with pLV-HCV4a C–P7 or pWPXL control plasmid by the calcium phosphate precipitation method. **a** 48 h post-transfection, transfected cells were immune-stained for the HCV Core protein using the HCV core antigen mouse monoclonal antibodies (C7-50) and the goat anti-mouse Alexa Fluor 555 secondary antibodies. The nuclei of cells were counter-stained with the Hoechst 33342 blue stain. Untransfected cells were used as a negative control for the expression of HCV proteins. **b** EGFP-expressing plasmid (pWPXL) was used as a positive control for fast assessing the successful transfection by the detection of EGFP fluorescence

To validate the efficacy of the engineered lentiviral transfer plasmid in driving the expression of HCV4a structural proteins, HEK293T cells were transfected with either control pWPXL or pLV-HCV4a C–P7 plasmids via the calcium phosphate precipitation method. Subsequently, the expression of HCV genes was investigated by examining the HCV core protein expression in the transfected cells by conducting the immunofluorescence (IF) analysis 48-hours post-transfection. Immunostaining for the core protein confirmed the capability of the constructed pLV-HCV4a C–P7 plasmid to efficiently express the core protein in the transfected cells (Fig. 2a). This finding provided evidence supporting the functionality of the engineered plasmid and its capacity to facilitate the expression of HCV4a structural proteins in the mammalian cells. The control EGFP-expressing lentiviral transfer plasmid (pWPXL) was used in parallel as a control to monitor the successful transfection by the fast detection of EGFP fluorescence in the pWPXL-transfected-cells (Fig. 2b).

### Production of functional Lentivectors (LVs) encoding HCV structural genes, HCV4a-Lentiviruses

**Fig. 3:**
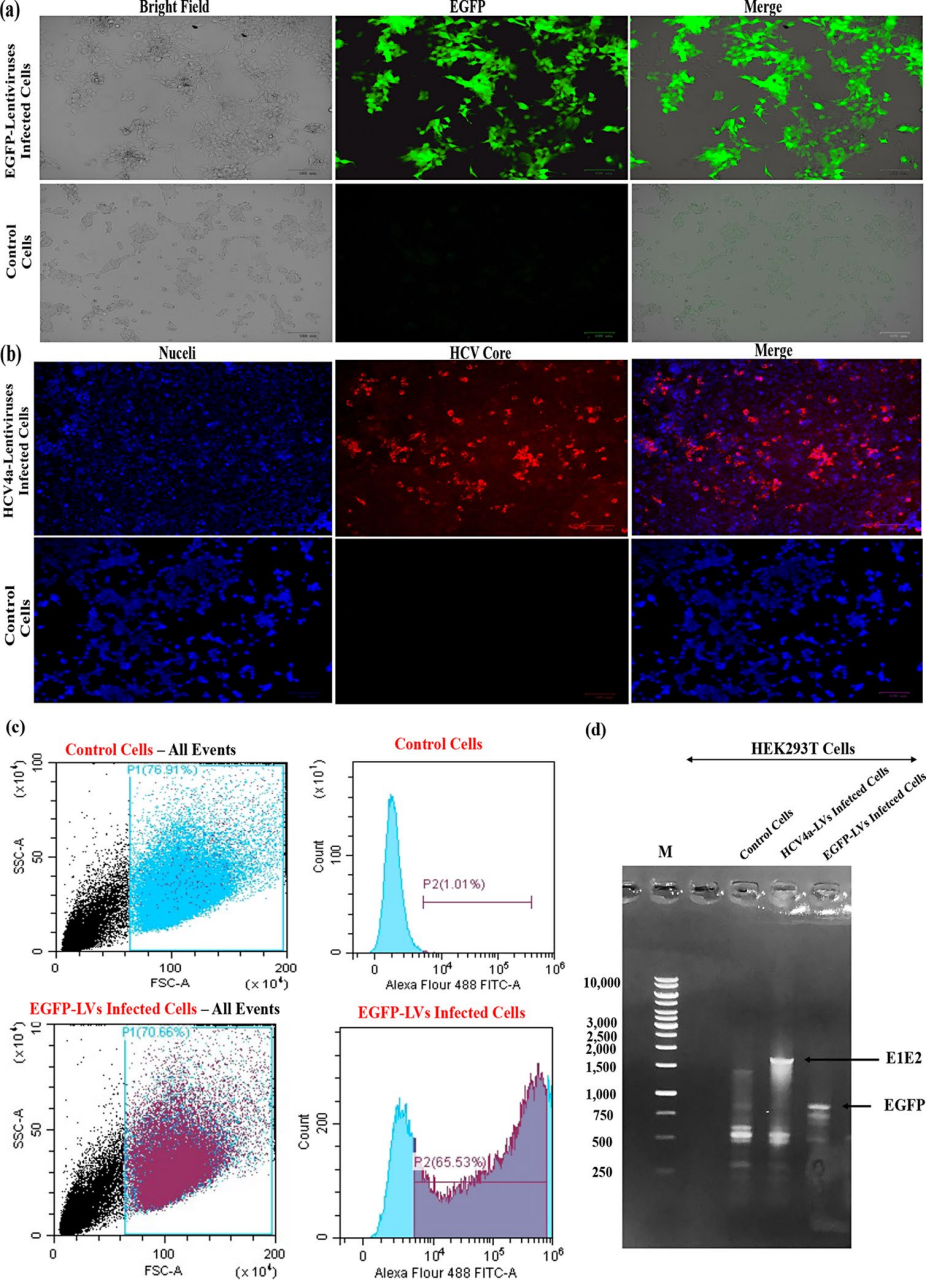
Production of functional lentivectors encoding HCV4a structural genes. **a** Production of functional EGFP-lentivectors, EGFP-LVs. HEK293T cells were co-transfected with the three lentiviral plasmids, pWPXL, psPAX2 and pMD2G. The produced EGFP-LVs were collected 48, 72 and 96 h post-transfection, centrifuged, filtered and used to infect cells. The expression of EGFP in the infected cells was investigated 48 h post-infection using the Zoe Fluorescent Cell Imager (BioRad, USA). Non-infected cells were used as a negative control for the EGFP expression. **b** Production of functional HCV4a-lentivectors (HCV4a-Lentiviruses). HEK293T cells were co-transfected with pLV-HCV4a C–P7, psPAX2 and pMD2G. HCV4a-Lentiviruses -containing medium was collected, centrifuged, filtered and used for transduction of fresh HEK293T cells. 48 h post-transduction, the expression of HCV core gene in the infected cells was examined by the immunostaining using the HCV core antigen mouse monoclonal antibodies (C7-50) and the goat anti-mouse Alexa Fluor 555 secondary antibodies. The nuclei of cells were counter-stained with the Hoechst 33342 blue stain. Non-transduced cells were used as a negative control. **c** The titration of EGFP-LVs was conducted by the flow cytometric analysis of EGFP-expressing cells that were infected with the harvested EGFP-LVs-containing medium. On the top side, the flow cytometric analysis of non-infected control cells (negative control), while on the bottom side, HEK293T cells were infected with EGFP-LVs and the number of EGFP-expressing cells was counted by the flow cytometry using Alexa Fluor 488 FITC-A channel for detecting the fluorescence of EGFP protein. P2 represents the percentage of EGFP-expressing cells compared to the total number of cells. **d** The integration of HCV genes into the genome of infected cells. The genes integration was investigated by isolation of total genomic DNA from normal, HCV4a-LVs, and EGFP-LVs infected cells. The isolated DNA was subjected to PCR analysis to examine the presence of HCV or EGFP genes in the DNA samples using specific primers, GT4-E1–F and GT4-E2–R (Table 1) for detection of 1665 bp DNA fragment of HCV4a E1E2. While the integration of EGFP ORF was investigated using vector-specific primers, pWPXL-Seq-For and pWPXL-Seq-Rev to amplify a DNA fragment of about 1011 bp comprising the EGFP ORF. The gel is showing the presence of both HCV E1E2 and EGFP bands in the total genome of cells suggesting their integration in the infected cells

Initially, to optimize the Lentiviruses, LVs, production and infection protocols, the lentiviral control vector encoding EGFP was generated through the co-transfection of HEK293T cells with three lentiviral plasmids, the lentiviral transfer, packaging, and envelope plasmids, pWPXL, psPAX2 and pMD2G, respectively. The produced EGFP-lentivectors (EGFP-LVs) were harvested and used in cells infection. The efficiency and titre of produced EGFP-LVs were assessed by infecting HEK293T cells with serially diluted virus-containing medium collected from the transfected cells. The infection results demonstrated that cells infected with EGFP-lentiviral vectors exhibited a robust expression of EGFP protein, confirming the successful production of functional EGFP-LVs and the efficient infection with these lentivectors (Fig. 3a). Subsequently, lentiviruses (LVs) encoding the HCV4a Core, E1, E2 and P7 genes were produced in HEK293T by co-transfecting the cells with the constructed lentiviral transfer plasmid, pLV-HCV4a C–P7, along with the packaging and envelope plasmids. The secreted HCV4a-Lentiviruses in the medium of transfected cells were harvested, purified and used to infect new normal HEK293T cells as target cells. The immunostaining of the HCV4a-Lentiviruses-infected cells for detecting the expression of HCV core protein demonstrated the successful expression of core protein in the infected cells. These results proved the generation of functional HCV4a-Lentiviruses capable of infecting HEK293T cells and expressing HCV genes (Fig. 3b). The titration of the EGFP-LVs production was conducted by the flow cytometry analysis of LVs-infected cells which demonstrated the capacity of lentiviral-based system to infect 65.5% of the total cells and produce a titre of 6 × 10^6^ transducing units per millilitre (TU/ml) (Fig. 3c). While the number of produced HCV4a-Lentiviruses was titrated by infecting the cells with serial dilutions of the lentivirus-containing medium and counting the number of the HCV core-expressing cells after immunostaining with the HCV specific anti-core antibodies. The obtained data confirmed the production of HCV4a-Lentiviruses of 5–6 × 10^6^ TU/ml comparable to the titre of EGFP-LVs.

Since lentiviruses (LVs) integrates the genes into the genome of infected cells as part of their life cycle, we investigated the presence of EGFP and HCV structural genes in the genome of the infected cells by the PCR analysis of the genomic DNA of the infected cells to confirm these integrations. The total genomic DNA was isolated from EGFP and HCV4a-Lentiviruses -infected cells and subjected to a PCR assay using specific primers to detect EGFP or HCV E1E2 genes (Fig. 3d). The results confirmed the presence of EGFP and HCV E1E2 genes in the genomes of infected cells but not in the genome of the control uninfected cells, indicating that the generated lentiviral vectors successfully delivered and integrated the HCV target genes (Core, E1, E2 and P7) into the genome of host cells for persistent protein expression.

### Establishment of a stable cell line for persistent expression of HCV4a structural proteins

**Fig. 4:**
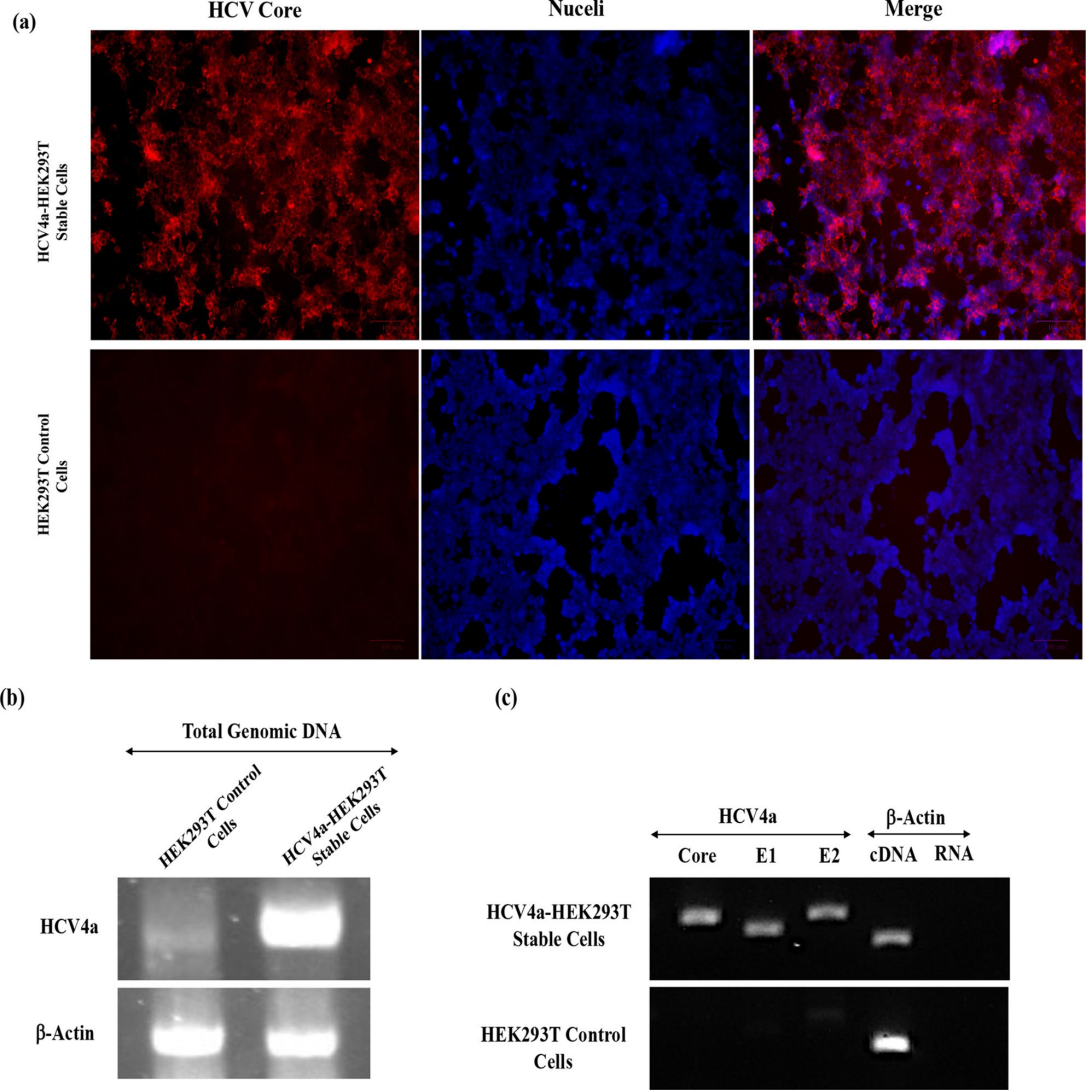
Establishment of HCV4a-HEK293T cell line for stable expression of HCV4a structural proteins. **a** Development of HCV4a-HEK293T stable cells. To develop a stable cell line for stable expression of HCV structural proteins, HEK293T cells were cultured and infected with the HCV4a-Lentiviruses. To isolate and enrich the HCV4a-HEK293T stable cells, the infected cells were maintained in medium containing puromycin (1 μg/ml) for 4–5 days to get rid of the uninfected cells before replacing the medium with fresh complete medium without the antibiotic for cells recovery. The expression of HCV core protein in the stable cells was investigated by IF using the HCV core antigen mouse monoclonal antibodies (C7-50) and the goat anti-mouse secondary antibodies conjugated with Alexa Fluor-555. Nuclei were counter-stained with the Hoechst 33342 blue stain. Un-infected cells were employed as a negative control for the core expression. **b** The integration of HCV genes into the genome of the established HCV4a-HEK293T stable cells. The total genomic DNA was isolated from the control and stable cells and subjected to the PCR analysis for detection of 1396 pb band of HCV4a Core-E2 genes using specific primers, HCV4a-RT-core-F and HCV4a-RT–E2–R. The β-actin gene was amplified using human β-Actin-F and Human β-Actin-R primers as a control. **c** Expression of HCV structural genes by stable cells. The mRNA expression of HCV integrated genes, Core, E1 and E2, in the HCV4a-HEK293T stable cells was examined by the isolation of the total RNA from normal and stable cells and used as templates to synthesize cDNA for both samples. The cDNA was subjected to the PCR analysis using specific primers for HCV4a Core, E1 and E2. The PCR for the β-actin gene from both cDNA samples was used as a control for the gel loading, while the PCR of β-actin gene using the RNA of both samples as a template was used as a control for detecting the DNA contamination in the RNA samples

To establish a stable cell line capable of constitutively expressing HCV structural proteins and producing virus-like particles for HCV genotype 4a (HCV4a-VLPs), HEK293T cells were transduced with HCV4a-Lentiviruses encoding HCV Core, E1, E2, and P7 as target cells. Following the transduction, infected cells demonstrated the expression of the HCV genes as indicated by the IF analysis and HCV genes integration as illustrated in Fig. 3b and d, respectively. Subsequently, cells expressing HCV genes were selectively enriched by culturing them in puromycin-containing medium for many days as a selection marker to make a pressure on cells for isolating only the HCV4a-expressing cells, HCV4a-HEK293T. Antibiotic-surviving cells were then maintained in a standard medium for further expansion and amplification. The resulting HCV4a-HEK293T cell line was assessed for HCV core protein expression by the immunofluorescence assay, revealing that approximately most of the cells were expressing the HCV core protein, indicative of the high efficiency of infection and puromycin selection for cells expressing HCV structural proteins (Fig. 4a). The established HCV4a genes-expressing stable cells (HCV4a-HEK293T) were propagated in a complete normal medium afterward for further investigations.

Plasmid transfection was conducted exclusively in packaging cells to generate lentiviruses, which were subsequently purified and used to infect target cells (fresh normal HEK293T cells) in a separate stage. Since no plasmids were introduced into the target cells, contamination from episomal plasmids was unlikely. Moreover, the lentiviruses carry only RNA genomes, ensuring no DNA contamination from the lentiviral genome during the infection of target cells. As a result, the only source of HCV4a DNA in the target cells which could be amplified by the DNA-dependent polymerase in the PCR assay is the integrated HCV genes within the genome of the target cells. This can confirm that the HCV genes are stably integrated into the host genome and not present as episomal constructs, further supporting the integrity of the stable cell line developed in the study. Therefore, the integration of HCV4a structural genes into the genome of established HCV4a-HEK293T stable cells was investigated by the PCR analysis of target cells genome. The total genomic DNA was extracted from the normal and stable cells, and the presence of HCV genes in the genome of stable cells was detected using specific primers for β-actin as a control or for a DNA fragment encompassing part of Core-E2 genes of HCV genotype 4a (Fig. 4b). The PCR results confirmed the presence of HCV genes in the genome of constructed stable cells, indicating the successful integration of HCV structural genes into the genome of established HCV4a-HEK293T stable cells.

As part of their life cycle, lentiviruses integrate their genetic material (HCV genes) into the host genome. These replication-incompetent lentiviruses complete only one cycle of infection, lasting for few days post-infection. Once the integration occurs, lentiviral particles are no longer present, and the focus shifts to stable expression of HCV genes directly from the host genome. In stable cells, the mRNA for HCV genes is exclusively transcribed from the integrated genome. Consequently, when the total RNA is extracted, converted to cDNA, and analyzed by PCR, the presence of HCV gene sequences in the mRNA confirms their stable integration and expression in the host cells. To investigate this critical step, mRNA expression of the integrated HCV genes prior to translation, we extracted total RNA from the cells, converted it to cDNA, and subjected it to PCR analysis to confirm the active expression of HCV genes (Core, E1, and E2) at the mRNA level. The mRNA expression of HCV4a structural genes (Core, E1 and E2) in the stable cells was also confirmed using the PCR analysis. The total RNA was isolated from HEK293T control and HCV4a-HEK293T stable cells. The cDNA was synthesized from the extracted RNA and used as a template in a PCR reaction to detect the expression of HCV4a genes with gene specific primers (Fig. 4c). The PCR results confirmed the mRNA expression of HCV4a Core, E1 and E2 genes in the stable cells but not in the control cells suggesting the successful mRNA expression of HCV4a integrated genes in the constructed HCV4a-HEK293T stable cells. β-actin cDNA was used as a positive control for the PCR reaction, while β-actin RNA served to confirm that the RNA preparation for the samples was free from genomic DNA contamination. This ensured that the PCR amplification was carried out exclusively using cDNA as the template, eliminating the possibility of interference from genomic DNA.

### Efficient generation of HCV4a-VLPs by the HCV4a-HEK293T stable cells

**Fig. 5:**
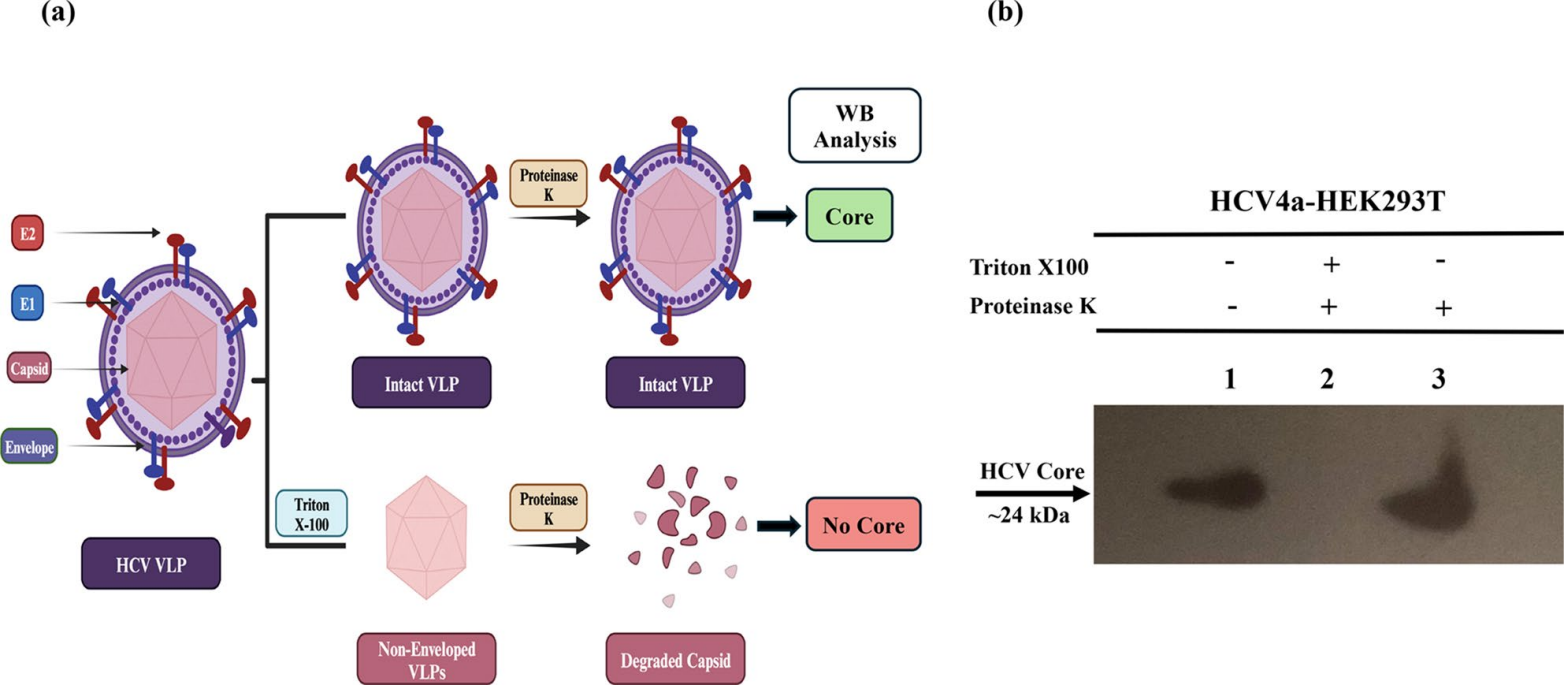
Production of two-layers enveloped VLPs by HCV4a-HEK293T stable cells. **a** A schematic diagram showing the principal of proteinase K protection assay. The structure of HCV-VLPs contains a central capsid of core protein surrounded by an outer lipid-bilayer originated from ER-derived membranes named the envelope and contains the HCV E1 and E2 glycoproteins. Because the VLPs contain both inner protein and outer lipid layers, selective solubilization of the outer envelope layer by Triton-X100 and the western blotting analysis for the presence or absence of the HCV core protein after Proteinase K digestion can be used to study the VLPs assembly process. This diagram was created with BioRender.com. **b** The assembly and envelopment of HCV4a-VLPs in the developed HCV4a-HEK293T stable cells. Enough number of HCV4a-HEK293T stable cells were cultured and harvested. The cell lysate was prepared and subjected to the treatment with either proteinase K alone or in combination with the detergent, Triton X100. The cell lysate was divided into three equal parts in separate tubes for the following different treatments; (1) Tube 1 for untreated cell lysate, (2) Tube 2 for the treatment with Triton-X100 followed by Proteinase K treatment of the cell lysate to cleave the core protein after dissolving the envelope with Triton X100, (3) Tube 3 for the treatment of cell lysate with only Proteinase K where the core is protected within the outer lipid layer of the envelope of the HCV4a-VLPs. After the treatments, all lysates were mixed with SDS-loading dye and subjected to the SDS-PAGE and WB analysis for detection of core protein using the HCV core antigen mouse monoclonal antibodies (C7-50) and goat anti-mouse IgG (H+L) secondary antibodies, HRP

The HCV virion particle comprises an inner capsid layer of the viral core protein within an outer envelope of an ER-derived membrane lipid bilayer and the viral E1 and E2 glycoproteins. The targeted disruption of these two layers and subsequent analysis to detect the presence or absence of resultant viral components can serve to investigate the virion assembly process (Fig. 5a). To take this advantage, the proteinase K protection assay was conducted to examine the production of enveloped VLPs by the engineered HCV4a-HEK293T stable cells (Fig. 5b). The assay results indicated that most of the HCV core protein was protected from proteinase k degradation in absence of the detergent, while it was undetectable in the presence of the detergent which dissolved the outer lipid layer of the envelope of the putative VLPs. These results revealed the presence of two-layered enveloped virus-like particles for the HCV genotype 4a, HCV4a-VLPs, an outer lipid envelope surrounding an inner capsid protein layer, which protected the core protein from the degradation by proteinase K. The assay results and the detection of the core protein band at 24 kDa indicated the successful processing of the Core-E1–E2–P7 single polyprotein encoded by the single ORF which was inserted into the host cell genome into individual proteins able to oligomerize and self-assemble into VLPs. Additionally, the nearly equal amounts of core protein in samples 1 and 3 in the western blotting after proteinase K treatment in Fig. 5b suggested that most of the core protein was successfully assembled into VLPs and protected from the proteinase K degradation by the surrounded lipid envelope.

### The generated HCV4a-VLPs exhibited physical properties comparable to the HCV mature virus

**Fig. 6:**
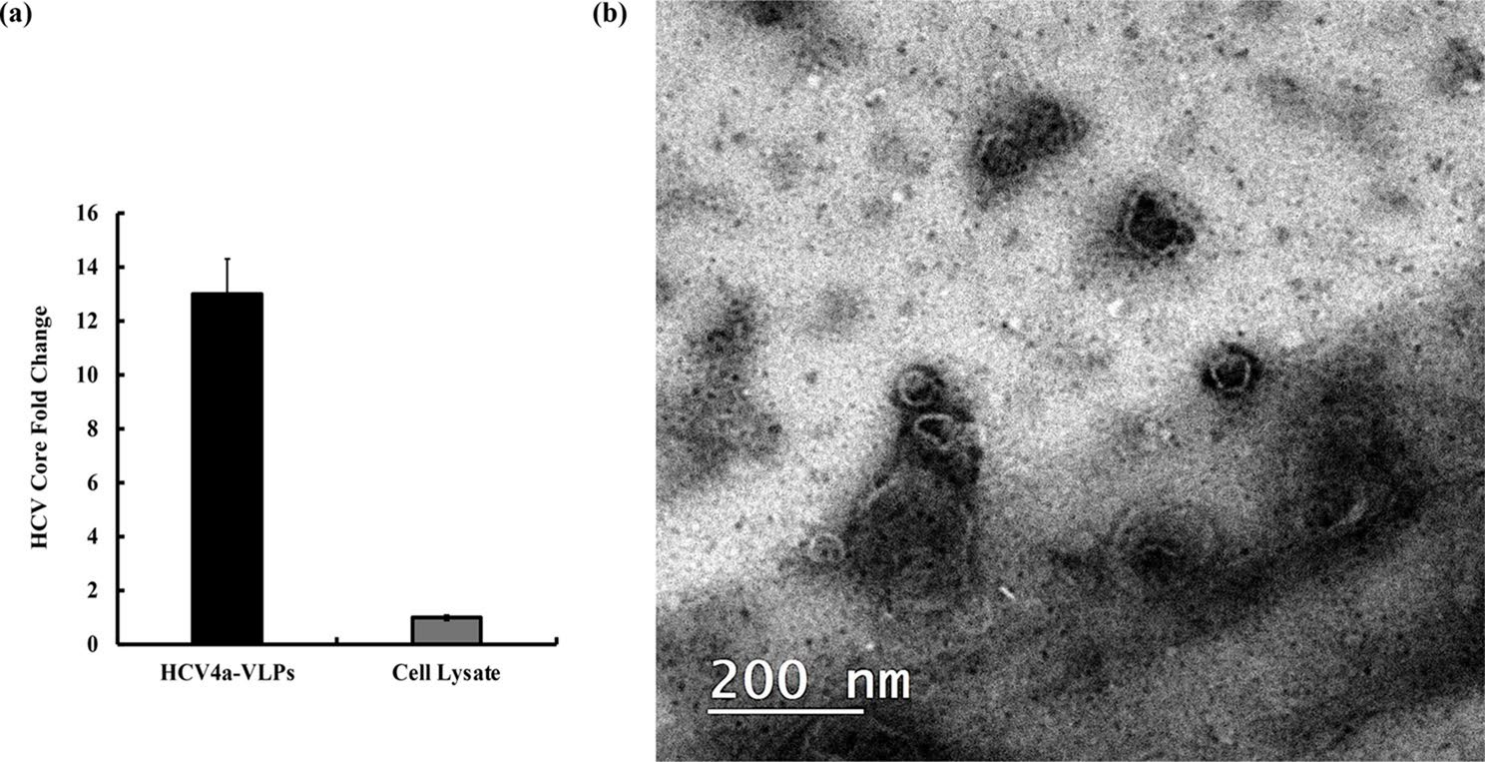
The generated HCV4a-VLPs exhibited physical properties comparable to HCV native virus. HCV4a-HEK293T stable cells were cultured and harvested for the preparation of cell lysate. The HCV4a-VLPs were isolated and purified from the cleared lysate by the ultracentrifugation on 30% sucrose cushion for 2 hours at 40,000 rpm at 4^o^C. The isolated HCV4a-VLPs were resuspended in PBS and examined by ELISA and TEM assays. **a** An ELISA assay for detecting the presence of HCV4a-VLPs in the pellet was conducted by detection of HCV core protein in the purified VLPs. The ELISA results confirmed the enrichment of the HCV core in the virus pellet after the isolation of VLPs by ultracentrifugation suggesting the successful formation and isolation of HCV4a-VLPs. The data are represented as mean ± SD of three replicates **b** The purified VLPs were investigated by the negative-staining TEM analysis. The results of TEM analysis revealed the presence of spherical VLPs particles of 60–65 nm average size similar to the shape and average size of the previously reported HCV native virus

To isolate and characterize HCV4a-VLPs produced by the established HCV4a-HEK293T stable cell line, a sufficient quantity of the stable cells was cultured and harvested for preparation of cell lysate. The HCV4a-VLPs were then isolated from the cleared cell lysate through the ultracentrifugation on a 30% sucrose cushion and resuspending the pellet containing HCV4a-VLPs in PBS. Following this partial purification, an ELISA assay for the core protein was carried out to investigate the enrichment of HCV4a-VLPs in the pellet isolated from stable cells lysate (Fig. 6a). The results indicated the presence of 13-fold increase in the mount of HCV core protein in the isolated VLPs over the stable cell lysate suggesting the efficient production and isolation of VLPs by the ultracentrifugation on sucrose cushion. To further confirm the successful generation and isolation of HCV4a-VLPs and investigate their physical characteristics, the isolated HCV4a-VLPs were examined using the Negative-Staining Transmission Electron Microscopy (TEM). The results of TEM analysis confirmed the presence of intact spherical VLPs with an average size of 60–65 nm, closely resembling the shape and dimensions of mature HCV virion particles (Fig. 6b). These VLPs structures were not found in the control cells. The characterization of HCV4a-VLPs by TEM highlighted the successful production and isolation of structurally accurate HCV4a-VLPs from the stable cell line that have the same morphology of the native HCV virus suggesting their potential for use as an HCV vaccine candidate.

### Long-term stability and persistent expression of HCV proteins by the established HCV4a-HEK293T cells

**Fig. 7:**
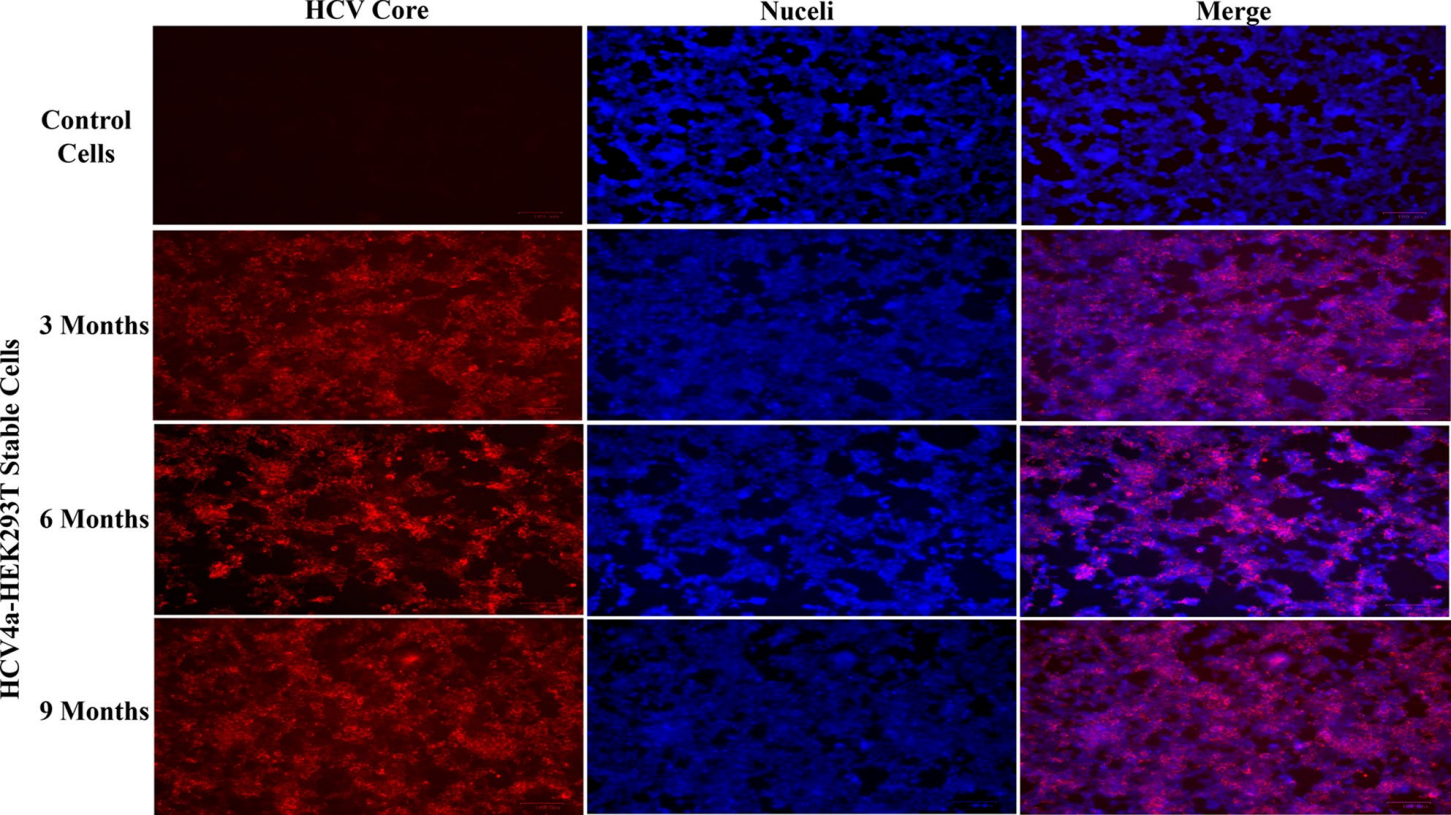
Long-term stability and persistent expression of HCV proteins by the established HCV4a-HEK293T cells. To evaluate the capability of stable cells for consistently expressing HCV genes over an extended period and generations, HCV proteins expression was assessed using the immunofluorescence staining of the Core protein. HCV4a-HEK293T cells were cultured and serially passaged in complete DMEM medium. At 3, 6, and 9 months, the expression of the Core protein was examined using HCV core antigen mouse monoclonal antibodies (C7-50) and goat anti-mouse secondary antibodies conjugated with Alexa Fluor-555. Nuclei were counterstained with the Hoechst 33342 dye. The results demonstrated that the cultured stable cells consistently expressed the HCV Core protein throughout the examined period, indicating the stability and persistence of protein production in the developed stable cells. This sustained expression is attributed to the integration of HCV genes into the host cell genome, allowing the genes to be passed on to subsequent generations

To verify the cell line’s ability to maintain its functional capacity for consistent expression of HCV proteins to produce VLPs reliably over extended periods, the expression of the HCV Core protein was monitored over a period of 9-months and sub-culturing of several generations following the establishment of the stable cell line (Fig. 7). The results confirmed that all cultured HCV4a-HEK293T stable cells maintained the viability and ability to express the HCV Core protein for the entire duration of the 9 months post-establishment suggesting the persistent production of HCV4a proteins and HCV4a-VLPs by these cells over multiple generations. This stability is achieved through the integration of HCV genes into the host cell genome, ensuring that these genes are faithfully passed on to daughter cells during subculturing. This indicates that the engineered stable cells are suitable for large-scale production of HCV structural proteins and VLPs-based vaccine.

## Discussion

Egypt has historically faced a high burden of HCV infections with the highest prevalence rate globally [5]. Without a prophylactic vaccine to stop viral transmission and reduce new infections, the elimination of HCV in Egypt may remain unattainable [8, 49]. Consequently, the disease will continue to negatively impact the Egyptian population and economy [6–9, 50]. HCV has 8 genotypes and 96 subtypes distributed all over the world [23, 24]. The significant sequence diversity of these genotypes and subtypes poses a challenge to developing a universal vaccine that is effective against all the circulating strains. The development of VLPs-based vaccine for HCV could meet the need to develop a universal vaccine for all HCV strains by presenting multiple antigens in their native conformation with the key conformational neutralizing and HCV-specific T cell epitopes [29, 38, 49]. In addition, as the HCV genotype 4 is the most dominant genotype in Egypt which represents 90% of total infections, the focus on developing a VLPs-based vaccine generated from the sequence of HCV genotype 4 could effectively target most of HCV infections in Egypt and control the viral transmission, thereby circumventing one of the primary challenges in HCV vaccine development [41, 49]. HCV genotype 4 has been under-researched and has received limited attention in the preclinical studies, particularly in the virus biology research and vaccine development. This is largely due to the geographical and socioeconomic context of the regions most affected like the Middle East, North Africa, and sub-Saharan Africa.

The HCV Core protein is highly conserved across genotypes, making it an excellent target for vaccine development. It has the ability to elicit robust T-cell responses [51, 52]. Similarly, the E1 and E2 glycoproteins play crucial roles in viral entry and immune recognition, forming E1E2 heterodimers on the virus surface with key neutralizing epitopes capable of inducing broadly neutralizing antibodies [53, 54]. E1 contributes to membrane fusion and contains immunogenic sites that can elicit neutralizing antibodies, while E2 features conformational epitopes critical for viral attachment to host receptors. Each protein plays a critical role in mimicking the structure and functions of the native virus, which is important for vaccine development and immunological studies [49, 55]. The p7 protein is a small, hydrophobic protein crucial for HCV assembly and infectivity, playing key roles in ion channel activity, virion assembly, stability, and immunogenicity [56, 57]. It forms ion channels (Viroporins) in membranes, maintaining the ionic environment essential for virion assembly and maturation, while also interacting with the core and glycoproteins to facilitate infectious particle production [58–61]. Additionally, p7 enhances the structural stability of the virus by ensuring proper envelope formation and integrity [60]. Though less immunogenic than other HCV proteins, it can stimulate cellular immunity, making it a potential target for vaccine development [56]. Therefore, the expression of HCV Core, E1, E2 and P7 in the form of VLPs in mammalian cells mimicking the native virus, will display multiple potential antigens with the key neutralizing and conformational epitopes required for induction of strong humoral and cellular immune responses overcoming one of the main challenges for the development of HCV vaccine development [41].

Lentiviral-based technology is a powerful tool for generating stable cell lines for the expression of vaccines, offering several advantages due to its efficiency, versatility, and durability. Lentiviruses are retroviruses capable of integrating their genetic material into the host genome, enabling long-term and stable expression of transgenes. This feature makes lentiviral vectors ideal for applications requiring sustained production of proteins, such as recombinant vaccine antigens. Unlike transient expression systems, lentiviral-based methods ensure consistent protein production across multiple cell generations, which is critical for scaling up vaccine manufacturing. Moreover, lentiviral vectors can efficiently transduce both dividing and non-dividing cells, broadening the range of host cells that can be used for vaccine production [62].

The process typically involves engineering a replication-deficient lentiviral vector carrying the genes of interest, which is then transduced into the host cell line. Once integrated, the transgenes remain stably expressed under the control of strong promoters, ensuring high levels of target protein expression. This system is particularly advantageous for producing complex or glycosylated proteins like HCV glycoproteins, E1 and E2, as it allows to select host cells that best support post-translational modifications. These properties make lentiviral vectors a good choice for the delivery of HCV structural proteins, Core, E1, E2 and P7 into the target cells genome for VLPs development, ensuring efficient, scalable, and reproducible production of high-quality VLPs as a potential vaccine candidate for HCV.

**Fig. 8:**
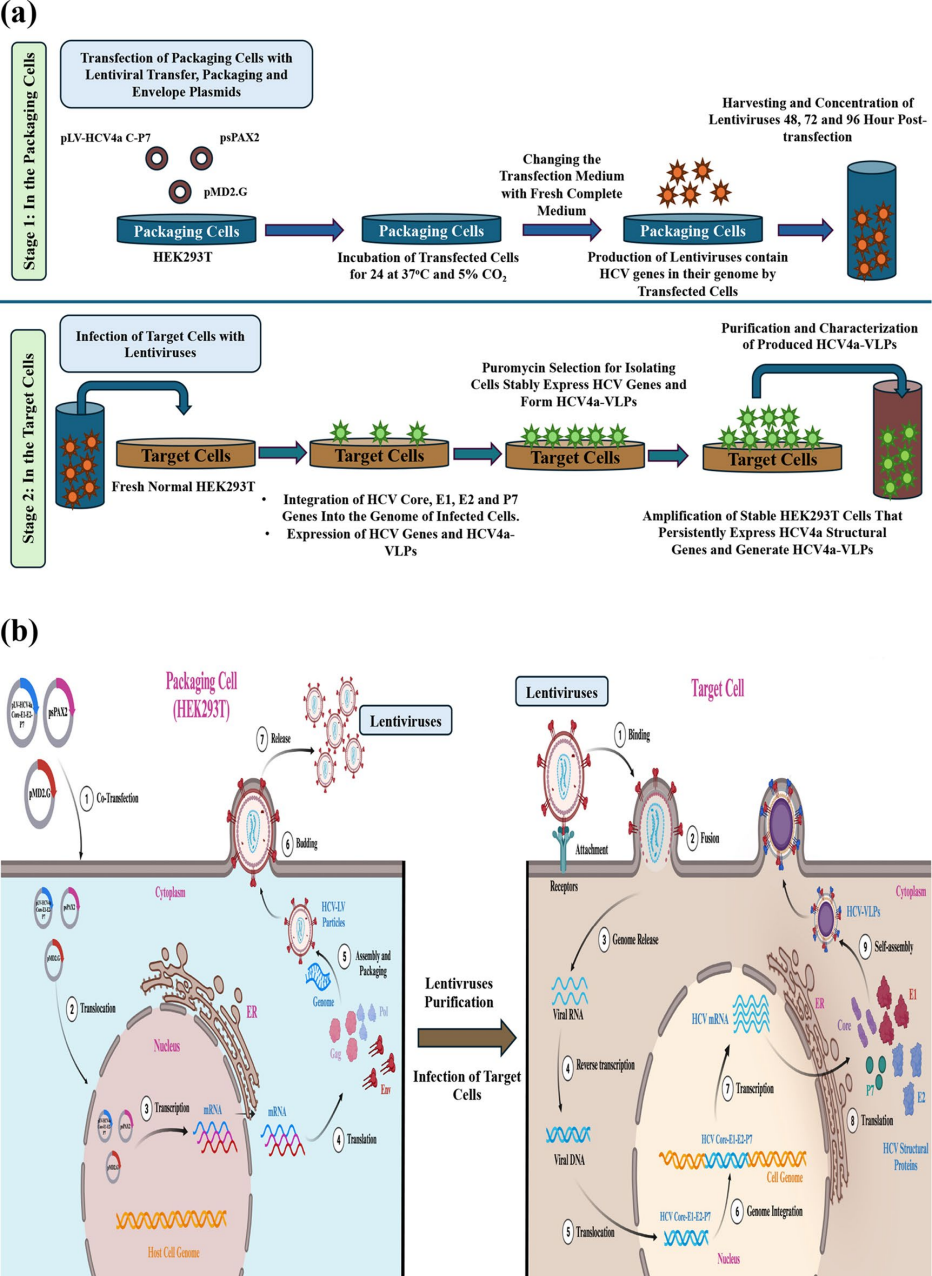
A schematic representation summarizes the establishment of HCV4a-HEK293T stable cells for persistent production of HCV4a-VLPs. **a** A workflow showing the in vitro generation of HCV4a-HEK293T stable cells that was carried out on two stages. The first stage (Top Panel) was performed in the packaging cells, HEK293T, to produce lentivectors carrying HCV4a structural genes (HCV4a-Lentiviruses). The second stage (Bottom Panel) was conducted in the target cells, HEK293T, by the transduction of target cells with the produced HCV4a-Lentiviruses to insert HCV4a four genes, Core, E1, E2 and P7 into the genome of the target cell for stable expression of VLPs. **b** A schematic diagram showing the in vivo lentiviral-based strategy for stable cells development. In the packaging cells, Left Side (1) Cells were co-transfected with three lentiviral plasmids, transfer (pLV-HCV4a C–P7), packaging (psPAX2) and envelope (pMD2.G) plasmids by the calcium phosphate precipitation method. (2, 3) Transfected plasmids were translocated to the nucleus and transcribed into mRNAs that were transported to the cytoplasm for the translation. (4, 5) The mRNAs were translated into lentiviruses components that can build the complete functional lentiviruses with a genome encoding HCV structural genes. (6, 7) The assembled HCV4a-lentiviruses exit the cells and released to the medium. The lentiviruses were purified, concentrated and used to infect new normal HEK293T cells as target cells. In the target cells, Right Side, the stable cell line was generated for persistent expression of HCV4a structural proteins, Core, E1, E2 and P7 and production of intact enveloped VLPs for HCV genotype 4a. (1, 2) The target cells were infected with the harvested HCV4a-Lentiviruses by binding to cell surface receptors and the fusion with the cell membrane for internalization. (3, 4, 5) The lentiviruses RNA genome was released into the cytoplasm and reverse transcribed into viral DNA which was translocated to the nucleus [87]. In the nucleus, the viral DNA was inserted and integrated into the genome of the target cells. (7, 8) The integrated HCV genes were transcribed into mRNAs of the HCV4a structural genes which were translocated to the cytoplasm for the translation on the surface of the ER. (9, 10, 11) The translated HCV proteins oligomerized and self-assembled into HCV4a-VLPs mimicking the HCV native virus. This diagram was created with BioRender.com

In the current study, we successfully employed the lentiviral-based technology to develop a stable mammalian cell line persistently expressing the viral structural proteins and generating VLPs for HCV genotype 4 overcoming a significant milestone in the development of a robust platform for production of a VLPs-based vaccine. The in vitro workflow and in vivo complete strategy of generation of VLPs and VLPs-producing cells for HCV genotype 4 in this study is summarized in Fig. 8a and b, respectively. The lentiviral-based vectors were efficiently employed to transfer and integrate the full ORFs sequence of HCV4a Core, E1, E2 and P7 proteins with the necessary expression elements and antibiotic selection marker into the HEK293T cells’ genome for consistent and reliable VLPs production. The HCV-integrated genes in the developed cells exhibited high expression level of HCV proteins, while the integrated puromycin-resistance gene was efficiently used to isolate the HCV4a-HEK293T stable cells. The development of stable cells expressing HCV structural proteins lays the groundwork for the subsequent VLPs production and characterization studies.

The results of proteinase K protection assay and TEM analysis provided compelling evidence for the oligomerization and self-assembly of the HCV Core, E1 and E2 proteins into enveloped VLPs by the established HCV4a-HEK293T stable cells. The proteinase K protection assay demonstrated that the produced VLPs have an inner capsid layer of the Core protein enclosed within an outer envelope made up of a lipid layer, which mimics the native HCV virus structure. The physical characterization of HCV4a-VLPs by the TEM analysis confirmed their spherical morphology with an average size of 60–65 nm, closely resembling the mature HCV virion particles, thus affirming their potential as a vaccine candidate [63–65]. The production of HCV4a proteins was validated over a 9-month period following the establishment of the stable cell line, providing strong evidence of the cells’ ability to consistently and persistently express HCV4a proteins and VLPs, which is crucial for large-scale and sustained vaccine production. Interestingly, the observed physical and morphological properties of the constructed HCV4a-VLPs are highly consistent with those reported for the isolated native virus, the infectious virus produced in the cell culture (HCVcc), and also with the VLPs produced for other HCV genotypes [65–67].

HCV was first identified in 1989 as the causative agent of non-A, non-B hepatitis (NANBH) [68]. The virus was observed as a spherical particle of 55–65 nm in diameter with an inner core of 30–35 nm [69]. After that, a significant breakthrough in HCV research occurred in 2005 with the development of a cell culture-based system for the production of HCV infectious virus (HCVcc) in vitro for genotype 2a strain which was isolated from a patient with a Japanese fulminant hepatitis, JFH1 [66]. This advancement allowed, for the first time, the functional investigation of the entire life cycle of HCV [66, 70, 71]. The first infectious HCV virus particles (HCVcc) produced in Huh7 for genotype 2a were shown to have a spherical shape with an average diameter of approximately 55 nm [66]. Later, HCV particles for different genotypes (1a, 1b, and 2a) were also produced in Huh7 and were found to own a round shape with about 50 nm diameter [72]. Eventually, chimeric infectious viruses derived from JFH1-based genotype 1 to 7 core-NS2 recombinants were also produced in cell culture with a similar morphology [73–75]. Taken together, the morphology of VLPs constructed in this study for genotype 4 is consistent with the previously reported morphology of the HCV native virus and the infectious virus of other genotypes produced in the cell culture, HCVcc.

Previous studies showed the presence of two types of VLPs that can be generated for HCV. The first type consists only of the core protein self-assembling into a capsid with approximately 30–50 nm in diameter [76–79]. The second type of VLPs comprises fully enveloped VLPs, with an inner capsid surrounded by an outer lipid bilayer envelope containing the viral glycoproteins E1 and E2, averaging 60–65 nm in size. Both forms of VLPs were investigated as potential vaccine candidates [80]. Various expression systems were used to generate both forms of the VLPs for some HCV genotypes, including bacteria, yeast, insect cells and mammalian cells. These VLPs demonstrated promising results in the induction of strong humoral and cellular immune responses in the preclinical studies [29, 38–40]. However, most of these studies were mainly focused on producing VLPs for HCV genotype 1, the most prevalent genotype globally. The morphological characters of VLPs produced for genotype 1a in Huh7 are closely similar to the constructed HCV4a-VLPs in our study. Interestingly, the vaccination of Balb/c mice with the purified HCV1a-VLPs generated broad neutralizing antibodies and strong HCV-specific CD8+ T-cell response highlighting the potentiality of VLPs as an effective vaccine against HCV [67]. Another study confirmed that the vaccination with recombinant genotype 1b VLPs, generated in insect cells, induced robust immune responses that were able to protect the chimpanzee from the chronic infection [81].

Efforts to produce VLPs for HCV genotypes 2 and 3 were also successful, with several studies demonstrating their ability to elicit robust immune responses [82]. Adenovirus-based system encoding HCV Core, E1 and E2 for genotype 3a was able to generate VLPs in the human hepatocellular carcinoma cells with an average size of 30–50 nm. A combined vaccination regimen of the produced VLPs with a recombinant adenovirus produced HCV-specific humoral and cellular responses [83]. The Core, E1 and E2 of four different genotypes (1a, 1b, 2a and 3a) were able to self-assemble when expressed in Huh7 cells into VLPs of similar morphology [82]. The vaccination of mice and pigs with a quadrivalent VLPs-based vaccine of these genotypes produced strong HCV-specific neutralizing antibodies and T cell responses [35–37]. However, there is limited research on producing VLPs for the other HCV genotypes like 4, 5, 6, 7 and 8 due to their restricted geographical distribution in developing countries [41].

Most previous studies on VLPs production for HCV relied on the transient expression of HCV structural proteins to generate VLPs. In contrast, in this study, the engineering of HEK293T cells for stable expression of these proteins and consistent VLP generation offers significant advantages [41]. The stable cells can continuously produce HCV4a proteins and VLPs without the need for constant reintroduction of the genetic material and repeating the transfection protocol which will save the time and cost of manufacturing, overcoming the main drawbacks of using mammalian cells as an expression system. HCV4a-HEK293T stable cells can be easily adapted to grow in suspension culture to increase the yield of VLPs for scaling up the production. Moreover, the development of HCV4a-HEK293T stable cells ensures consistent production of the VLPs, which can lead to a reproducible vaccine quality and efficacy.

The stable expression of VLPs in mammalian cells is increasingly explored technology to produce future vaccines due to its potential for producing more complex post-translational modifications and higher-fidelity VLPs. Using lentivirus-based vectors, HEK293T stable cells were developed for production of VLPs-based multivalent vaccine candidate against four different viruses, Zika virus, Chikungunya, Yellow fever and Japanese encephalitis [84] virus. Vaccination of Balb/c mice with these VLPs generated strong neutralizing antibodies. This stable cell line was adapted to grow in a suspension culture with the ability to secrete the VLPs to the medium [85]. Recently, HEK293 cells were also used to generate stable cell line for persistence expression of functional SARS-CoV-2 VLPs as a potential vaccine candidate [86].

The generated HCV4a-HEK293T as stable VLPs-producing cells could find a wide range of applications in various fields. Primarily, these cells can produce VLPs-based vaccine with potential immunogenic properties and high safety profile. The VLPs and VLPs-producing cells can be used for the development of diagnostic reagents and assays for HCV genotype 4 and similar genotypes. In addition, the produced HCV4a-VLPs can serve as a safe model for studying the virus entry, assembly and interactions without the need for handling the infectious virus. The future directions for this study will focus on scaling-up the production and assessing the immunogenicity of the generated VLPs. Additionally, studies will be conducted to determine the protective efficacy of the VLP-based vaccine in animal and using the In Vitro HCV models, with the potential for clinical trials.

## Conclusion

Although HCV genotype 4 is less well-known than other genotypes, it represents a significant public health challenge in regions where it is most prevalent like Egypt. By increasing the focus on genotype 4 through the vaccine research and development, it is possible to address the neglect of this genotype and move closer to the virus elimination. VLP-based vaccines offer a promising solution to challenges in HCV vaccine development. Their ability to elicit robust humoral and cellular immune responses, safety, adaptability to include multiple antigens, and displaying neutralizing and conformational epitopes make them strong candidates for future vaccines. In the present study, the successful establishment of a stable mammalian cell line capable of robustly expressing HCV structural proteins and generating non-infectious VLPs for genotype 4 for the first time could represent a significant advancement in vaccine development especially against HCV genotype 4. This approach not only ensures the production of VLPs-based vaccine that closely resemble the natural virus, but also allows for scalable and consistent vaccine manufacturing by the stable cells. In addition, the developed VLPs and HCV4a-HEK293T stable cells constitute a valuable non-infectious model for studying the HCV genotype 4 entry, assembly and morphogenesis. Additionally, the stable cells can be employed for the development of simple and cost-effective HCV diagnostic assays and reagents. Overall, the generated VLPs and the stable cell line could significantly reduce the initial costs and speed up the production timeline of VLPs-based vaccine. This work lays the groundwork for further investigations aimed at optimizing VLPs production and assessing their immunogenicity as a potential vaccine against HCV genotype 4.

## Data Availability

All relevant data, methods and materials are detailed in the manuscript.
